# Vessel Density and Structural Measurements in Primary Angle-Closure Suspect Glaucoma Using Optical Coherence Tomography Angiography

**DOI:** 10.1155/2020/7526185

**Published:** 2020-12-02

**Authors:** Yi Zha, Juanjuan Chen, Shuyu Liu, Jinfei Zhuang, Jianqiu Cai

**Affiliations:** ^1^Department of Ophthalmology, The Second Affiliated Hospital and Yuying Children's Hospital of Wenzhou Medical University, Wenzhou, Zhejiang 325027, China; ^2^Department of Ophthalmology, The First Affiliated Hospital of Jiangxi Medical College, Shangrao, Jiangxi 330000, China

## Abstract

**Purpose:**

To measure the macular retinal vessel density (VD) and peripapillary retinal nerve fiber layer (RNFL) in primary angle-closure suspects (PACS) by Angio-OCT to be compared with normal subjects.

**Methods:**

Primary angle-closure suspect patients and normal subjects were enrolled in this study. The demographic and clinical characteristics of all subjects, such as RNFL thickness, retinal vessel density, and ocular perfusion pressure, were compared.

**Results:**

No significant difference was found in both groups on age, sex distribution, intraocular pressure (IOP), and retinal vessel density. The PACS group exhibited significantly thicker RNFL thickness compared with the control group. The deep vessel density was negatively associated with age (*P* = −0.034), while IOP had negative association with ACD (*P* = −0.019). OPP was independently associated with RNFL (*B* = 0.334, *P* = 0.038) in the PACS group.

**Conclusions:**

OCTA showed significant thicker change on RNFL in the PACS group. Only OPP was independently associated with RNFL in the PACS group.

## 1. Background

Primary angle-closure glaucoma (PACG), the most common type of glaucoma in Asia, is one of the leading causes of eye blindness [1, 2]. IOP elevation and visual field loss are two of the most important glaucomatous damage on a PACG eye.

Mechanical compression and vascular insufficiency are known as two major mechanisms of glaucomatous damage such as optic neuropathy and vision field loss [3, 4]. The development of optical coherence tomography angiography (OCTA) has made it possible to detect vascular factor on glaucoma eyes. Evidences showed that retinal vessels had the autoregulation capacity following up with the fluctuation of IOP and blood pressure. The vessel density parameter might be useful in all stages of glaucoma. Even without detected visual field damage, OCTA could still find some retinal microvasculature change in PACG eyes [5, 6].

Previous studies have showed that changed macular vessel density happened earlier than structural and functional damage in glaucomatous eyes. Macular vessel density decreased early while peripapillary vessel density decreased late in glaucoma [5, 7–9]. The purpose of this study was to detect primary angle-closure suspect (PACS) patients' macular microvasculature and peripapillary retinal nerve fiber layer (RNFL) and compare them with the healthy control group. We hypothesize that the PACS stage might have microvascular or structural changes on eyes that play a role in the early pathogenesis of PACG.

## 2. Methods

This was a case-control study. This study was conducted by the Declaration of Helsinki and approved by the ethics committee of the 2^nd^ Affiliated Hospital of Wenzhou Medical University which belonged to Wenzhou Medical University. All of the participants involved in the study were required to sign written informed consent.

### 2.1. Patients

Consecutive PACS subjects were enrolled from outpatient clinic. Healthy subjects were collected from our Examination Center.

The inclusion criteria of all subjects were as follows: corrected distance vision acuity (subjective refraction) of ≤0.3 LogMar, refractive error of ≤±5.00 diopters (D) spherical and ≤±3.00 D cylindrical, and age between 50 years and 70 years.

The diagnostic criteria for PACS included both of the following: (1) a pigmented trabecular meshwork in the eye that was not visible for ≥180° under static gonioscopy; (2) an IOP lower than 21 mmHg, without glaucomatous neuropathy or peripheral anterior synechiae; (3) normal visual field and regular disc morphology [10]; and (4) the presence of a narrow-angle pupillary block component which was confirmed by an ultrasound biomicroscopy (UBM) examination, which was defined as anterior chamber depth (ACD) < 2.5 mm and peripheral anterior chamber depth (PACD) < 1/4CT. The healthy control had normal ACD and no history of taking medicine that may have influence on ocular blood flow.

The exclusion criteria of all subjects were as follows: any history of high IOP, any evidence of retinal or neurological disease, any history of intraocular surgery correlated with glaucoma, obvious refractive media opacity that might affect imaging quality, and those who could not cooperate with the examination and fixation losses.

All subjects enrolled in this study underwent a set of examinations including best corrected visual acuity (BCVA), autorefraction, slit lamp, gonioscope, IOP (Goldmann applanation tonometry), ultrasound biomicroscopy (UBM), visual field (Humphrey Field analyzer II, model 720i), optical coherence tomography (OCT), and systemic blood pressure. All subjects were required to rest in a seated position in a quiet room for 25 min before taking their systemic blood pressure and OCT images.

The mean arterial pressure (MAP) was calculated as the diastolic blood pressure plus one-third of the difference between the diastolic blood pressure and the systolic blood pressure (MAP = DBP + 1/3(SBP‐DBP)). The ocular perfusion pressure (OPP) was calculated as OPP = 2/3MAP‐IOP. Normal VF was defined as MD and PSD within 95% confidence intervals and a glaucoma hemifield test result “within normal limits.”

### 2.2. OCT Imaging Acquisition

**Figure 1:**
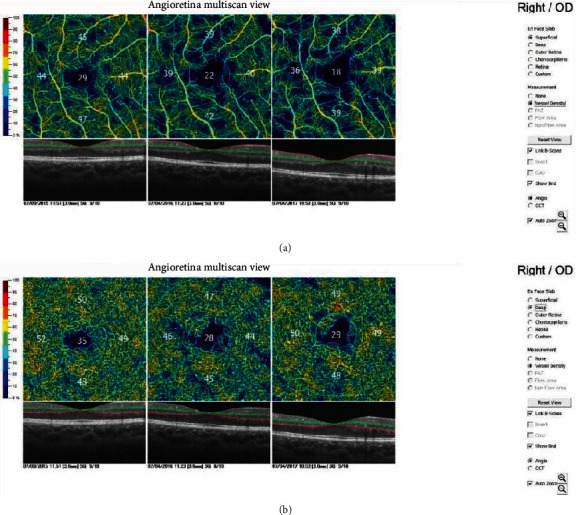
Angioretina multiscan (OCTA) showing superficial vessel density (a) and deep vessel density (b).

The same experienced examiner finished all OCT measurement (RTVue XR Avanti, Optovue, Inc., Fremont, CA) on nonmydriatic eyes. Only images of good quality of OCT were used for further analysis. The scans were obtained using the AngioVue software SD-OCT system. Two scan volumes with a 3.0 × 3.0 mm scanning area centered on the fovea were captured for each patient. The merged 3-dimensional OCT angiograms were produced using the AngioVue software and then exported for predefined retinal vessel density (VD) measurement. The superficial vessel density (VDS) and deep vessel density (VDD) data were collected for analysis. Superficial was predefined as ILM to IPL-10 *μ*m, while deep was predefined as IPL-10 *μ*m to OPL+10 *μ*m (Figure 1).

**Figure 2:**
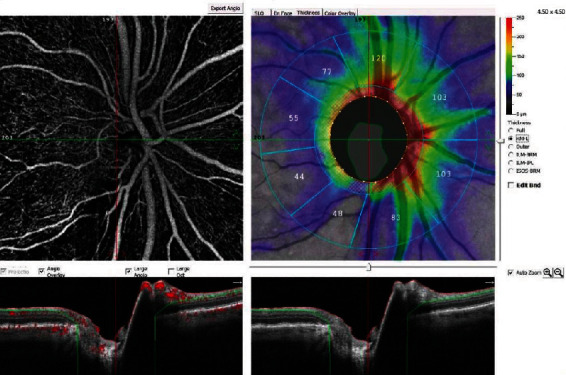
Angio-disc RNFL peripapillary thickness by OCTA.

For peripapillary RNFL analysis, the peripapillary region was defined by two rings of 2 mm and 4 mm centered on the disc center. AngioAnalytics was used for RNFL thickness measurement. RNFL thickness metrics within the 2-4 mm diameter Garway-Heath-based grid areas were available for review under the Thickness Tab of the OCT image on the Main Report screen for the RPC (Radial Peripapillary Capillaries) slab (ILM to NFL). The average RNFL thickness was recorded separately (Figure 2).

### 2.3. Statistical Analysis

SPSS software version 21.0 for Microsoft Windows (IBM Inc., Chicago, USA) was used for statistical analysis. All data were expressed as the mean ± standard error. Independent sample *t* and chi-squared tests were used to determine significant differences between the groups for continuous and categorical variables, respectively. The Pearson correlation test was used to detect the association between VD, RNFL thicknesses, IOP, and age. The multiple linear regression model was adjusted for OPP, VDD, VDS, SBP, DBP, IOP, and ACD compared with RNFL. Results with *P* values of less than 0.05 were considered statistically significant.

## 3. Results

43 eyes of 23 PACS patients and 29 eyes of 22 healthy controls were recruited in the end for the study. 4 PACS eyes and 3 healthy control eyes were excluded because of poor image quality. A total of 39 eyes of 20 PACS subjects and 26 eyes of age- and sex-matched healthy controls were included in the final analysis.

Age (*P* = 0.21) and sex (*P* = 0.079) were comparable between the PACS and healthy groups. The demographic and clinical characteristics of all subjects are listed in Table 1. There were statistically significant differences between two groups in terms of RNFL, ACD, and BCVA (*P* < 0.01), while there was no significant difference in IOP, VDD, VDS, OPP, SBP, and DBP between the PACS group and the control group.

In the PACS eyes, there was no significant correlation between VDS, RNFL, and IOP (*P* > 0.05). Correlation analyses showed that the VDD was negatively associated with age (*P* = −0.034), while IOP had negative association with ACD (*P* = −0.019). Multiple linear regression analysis using RNFL as the dependent variable and covariates (OPP, VDD, VDS, SBP, DBP, IOP, and ACD) as independent variables showed that only OPP was independently associated with RNFL (*B* = 0.334, *P* = 0.038) in the PACS group.

## 4. Discussion

In this study, we compared the demographic and clinical parameters in PACS eyes with those in healthy control eyes and found that VDD, VDS, and IOP were all similar in PACS patients and healthy controls. However, the RNFL by OCTA showed significant thicker change in the PACS group than normal controls. Only OPP was independently associated with RNFL in the PACS group.

The development of OCT has made it easy to detect glaucoma at early ages. In this research, 20 healthy subjects were enrolled first for evaluating the repeatability and reproducibility of OCTA in the RNFL, VDD, and VDS. The good agreement of intragroup and intergroup differences had made further results more reliable.

Previous researches have showed structure and vascular changes on PACG and POAG eyes as well [11–14]. PACG was considered to be a more IOP-independent disease. Tsai et al. [15] compared the affected eyes with their fellow eyes after an attack and found a thicker RNFL at 1 week and attributed it to the mild edema of optic nerve. Moghimi [16] demonstrated that after an APAC (acute primary angle closure) episode, vessel density decreased while RNFL thickness increased at first, followed by a subsequent decrease. Aung et al. [17] reported that RNFL thickness was found to decrease significantly from 2 to 16 weeks after APAC. Wang et al. [18] found a decreased VD at the peripapillary area but similar structural measurements such as RNFL thickness in APAC eyes. Most studies mentioned above had one disadvantage: the control group was the fellow PACS eyes. Since a PACS eye could in part be turned into APAC, it is not sure if there had already been changes on RNFL or VD in the PACS stage. Different results do exist in RNFL thickness. After a IOP spike occurring during the acute episode, VD and VF changed while RNFL remained the same or increased first and then decreased late. Two explanations were proposed: (1) RNFL was inclined to be changed by long-term chronic lesions but not acute lesion and (2) chronic change may have happened on RNFL in the PACS stage. In this study, the RNFL but not VD showed significant thicker change in the PACS group than normal controls, which implied that in the PACS stage, chronic change might have happened on RNFL while no changes happened on VD. According to our result, RNFL may be a more sensitive index for predicting the changes in the PACS stage.

Vascular factors played an important role in the development of glaucoma [14, 19, 20]. Jo et al. [21] found that the relationship between vascular and function characteristics was stronger than that between structure and function characteristics. Zhang et al. [22] followed 12 years of PACG eyes and found a significant correlation between vessel density and structure parameters.

Previous studies have showed the same glaucoma-diagnostic ability of macular VD as that of RNFL parameters. Yarmohammadi et al. [14] compared RNFL thickness and retinal vasculature parameters and found that OCTA vessel density had similar diagnostic accuracy to RNFL thickness for differentiating between healthy and glaucoma eyes. Zhu et al. [23] and Rao et al. [13] both found that PACG eyes had lower VD and RNFL thickness compared with healthy eyes, but the reduction in VD was greater in the peripapillary than parafoveal area. Retinal vessels in the parafoveal and peripapillary areas responded differently in blood pressure and oxygen pressure [24]. The retinal macular region was only supplied by the retinal artery, while the optic disc was supplied by the ciliary and retinal arteries. Retinal circulation was impaired only when IOP was elevated to the central retinal artery pressure. When IOP increased high enough, the disc and peripapillary choroid capillaries were obliterated and the retinal circulation was slowed [25, 26]. From another point of view, when IOP was elevated, the swelling of the optic disc and nerve would be more apparent than that of macular [27]. Therefore, it was quite reasonable to speculate that in the PACG stage, VD in the peripapillary area was influenced more severely than that of the macular area, whereas in the PACS stage, since IOP was always normal, the macular area may be more sensitive and vulnerable.

Kim et al. [7] speculated that macular SMD (superficial microvessel density) may be potentially useful in the clinical evaluation of early glaucoma. As far as we know, there was no other study that focused on PACS eyes to find any change on macular VD. In our study, VDD and VDS were chosen to be compared. We found that there was no significant difference in VDD and VDS between the PACS group and healthy eyes. Since PACS could be partly developed into the APAC stage, it was quite necessary to analyze the macular VD which may be more sensitive in the PACS stage. According to our result, it is postulated that in our PACS group, VD might be developed too slowly to be noticed in statistics.

In this study, only OPP was independently associated with RNFL. It was implied that during the PACS stage, OPP may influence more on RNFL before IOP changed.

There were several limitations in our research. First was the small sample size. Since no research was related to the database values of PACS patients for the OCTA, further studies with larger samples were needed. Second was that OCTA did not directly measure blood flow. The flow signal in OCTA comes from the motion of blood cells which may have error on reflecting perfusion status. Third, blood pressure condition was not strictly controlled.

## 5. Conclusions

In conclusion, VDD, VDS, and IOP were all similar in PACS patients and healthy controls. However, the RNFL by OCTA showed significant thicker change in the PACS group than normal controls. Only OPP was independently associated with RNFL in the PACS group.

## Figures and Tables

**Table 1:** The demographic and clinical characteristics.

	PACS	Healthy control	*P*
Age	61.79 ± 5.45	64.08 ± 7.98	0.210
Sex (M/F)^∗^	4/35	7/19	0.079
BCVA	0.03 ± 0.05	0.15 ± 0.14	0.000
VDD	48.17 ± 3.75	47.13 ± 2.94	0.238
VDS	46.39 ± 5.20	47.18 ± 2.54	0.418
OPP	48.45 ± 10.54	47.82 ± 6.67	0.768
SBP	126.91 ± 22.44	127.82 ± 14.12	0.841
DBP	74.97 ± 13.10	74.84 ± 9.22	0.963
IOP	13.07 ± 3.64	13.85 ± 2.54	0.349
ACD	2.07 ± 0.15	3.11 ± 0.18	0.000
RNFL	101.79 ± 9.20	92.92 ± 10.34	0.001

Data shown above are means ± standard deviations or numbers of subjects. ∗: *χ*^2^ test was applied. BCVA: best corrected visual acuity; IOP: intraocular pressure; OPP: ocular perfusion pressure; SBP: systolic blood pressure; DBP: diastolic blood pressure; ACD: anterior chamber depth; RNFL: retinal nerve fiber layer.

## Data Availability

The data is unavailable because the patients do not want their data to be released.
